# An update on SARS-CoV-2 immunization and future directions

**DOI:** 10.3389/fphar.2023.1125305

**Published:** 2023-03-09

**Authors:** Rashmi Rana, Ravi Kant, Tanya Kumra, Sneha Gupta, Devinder Singh Rana, Nirmal Kumar Ganguly

**Affiliations:** ^1^ Department of Research, Sir Ganga Ram Hospital, New Delhi, India; ^2^ Department of Nephrology, Sir Ganga Ram Hospital, New Delhi, India

**Keywords:** COVID-19, SARS-CoV-2, vaccine efficacy, herd immunity, reinfection, convalescent plasma therapy, Pfizer, Moderna

## Abstract

Millions of people have died as a result of SARS-CoV-2, which was first discovered in China and has since spread globally. Patients with SARS-CoV-2 infection may show a range of symptoms, including fever, coughing, and shortness of breath, or they may show no symptoms at all. To treat COVID-19 symptoms and avoid serious infections, many medications and vaccinations have been employed. However, to entirely eradicate COVID-19 from the world, next-generation vaccine research is required because of the devastating consequences it is having for humanity and every nation’s economy. Scientists are working hard to eradicate this dangerous virus across the world. SARS-CoV-2 has also undergone significant mutation, leading to distinct viral types such as the alpha, beta, gamma, delta, and omicron variants. This has sparked discussion about the effectiveness of current vaccines for the newly formed variants. A proper comparison of these vaccinations is required to compare their efficacy as the number of people immunized against SARS-CoV-2 globally increases. Population-level statistics evaluating the capacity of these vaccines to reduce infection are therefore being developed. In this paper, we analyze the many vaccines on the market in terms of their production process, price, dosage needed, and efficacy. This article also discusses the challenges of achieving herd immunity, the likelihood of reinfection, and the importance of convalescent plasma therapy in reducing infection.

## Introduction

Millions of people have died as a result of SARS-CoV-2, which was first discovered in China and has since spread globally (Wu et al., 2020; Zhou et al., 2020; WHO, 2022). Of the four different genera of the Coronaviridae family, i.e., alpha, beta, gamma, and delta-coronavirus, SARS-CoV-2 belongs to the beta genus. The characteristic features of the Coronaviridae family are a positive-sensed RNA virus enclosed by an envelope (Almeida and Tyrrell, 1967; Kapikian et al., 1969; Peiris et al., 2004; Van Der Hoek et al., 2004; Woo et al., 2005; Zaki et al., 2012). Since its discovery, the genome of the virus has undergone numerous modifications resulting in numerous mutant strains, including alpha, beta, and delta variants. According to the genetic sequence of the virus, which was first published in January 2020, SARS-CoV-2 has distinct characteristics, such as a strong affinity for the angiotensin-converting enzyme 2 (ACE2) receptor and a polybasic cleavage site at the S1/S2 spike junction that determines infectivity and host range (Nao et al., 2017; Andersen et al., 2020). SARS-CoV-2-infected patients can be asymptomatic or symptomatic and may show a number of symptoms, such as fever, cough, and shortness of breath. Occasionally, infected patients can also show symptoms including vomiting, diarrhea, and abdominal pain (Wang et al., 2020). Individuals who acquire pneumonia after COVID-19 infection show mottling and ground-glass opacity in chest X-rays (Zhu et al., 2020). Along with the primary target of the lungs, other organs of the body, such as the kidneys and liver, are also affected by COVID-19 infection (Renu et al., 2020). SARS-CoV-2 transmission occurs with high efficacy and infectivity, mainly through the respiratory route and primarily through droplet transmission (Han et al., 2020; Leung et al., 2020). At present, coronavirus is a dominating concern throughout the world. The severe effects of COVID-19 on humanity and the economy of every country require next-generation vaccine development to completely end this virus. Every non-profit organization and country in the world is attempting to fund vaccine companies to provide a vaccine development fund. Through valiant efforts by the scientific community, the first COVID-19 vaccine entered human clinical trials in 2020 (Thanh Le et al., 2020). However, the major issues in vaccine development are the absence of an animal model, the time-consuming process, and an unknown mechanism of pathogenesis (Mukherjee, 2020). Additionally, the continuous development of new genetic variants of SARS-CoV-2 is also an issue in generating an effective vaccine (Aljabali et al., 2020; Amawi et al., 2020; Shereen et al., 2020; Velavan & Meyer, 2020). Utilizing a small number of human trials, an ideal vaccine dosage and administration schedule should be established. Existing drugs for other viruses can also be examined for use as drugs for the COVID-19 virus (Dhama et al., 2020). Apart from vaccine development, there is also a need to check the time period for which antibodies are present in an individual after recovery, because the level of antibodies may relate to the probability of reinfection with the COVID-19 virus, and a great deal of research is ongoing to determine the probability of reinfection with the virus. Reinfection occurs when a person develops an infection once, recovers, and then becomes infected again, either with the same infectious agent or with a different variant (Yahav et al., 2021). The CDC states that recovered individuals must have at least one negative PCR test result for SARS-CoV-2. Reinfection of a patient can be immensely important because if reinfections are common, natural immunization will not be sufficient to confer herd immunity. Herd immunity is the indirect protection of susceptible individuals from the infection due to the presence of a large proportion of immunized individuals. Scientists are also working to determine the role of convalescent plasma therapy to treat or reduce the severity of COVID-19 infection. Convalescent plasma has already been tested for efficacy against other respiratory viruses. This therapy is hypothesized to initiate a temporary immune response against infectious virus particles and serve as a safeguard before the peak-level production of antibodies by the immune system of the infected individual (Luke et al., 2006; Mair-Jenkins et al., 2015; Arabi et al., 2016). In this article, we compare the different available vaccines in terms of their methods of production, cost, and effectiveness. Additionally, we discuss the potential for reinfection with the COVID-19 virus and how convalescent plasma therapy is used to treat infected people.

### SARS-CoV-2

**FIGURE 1:**
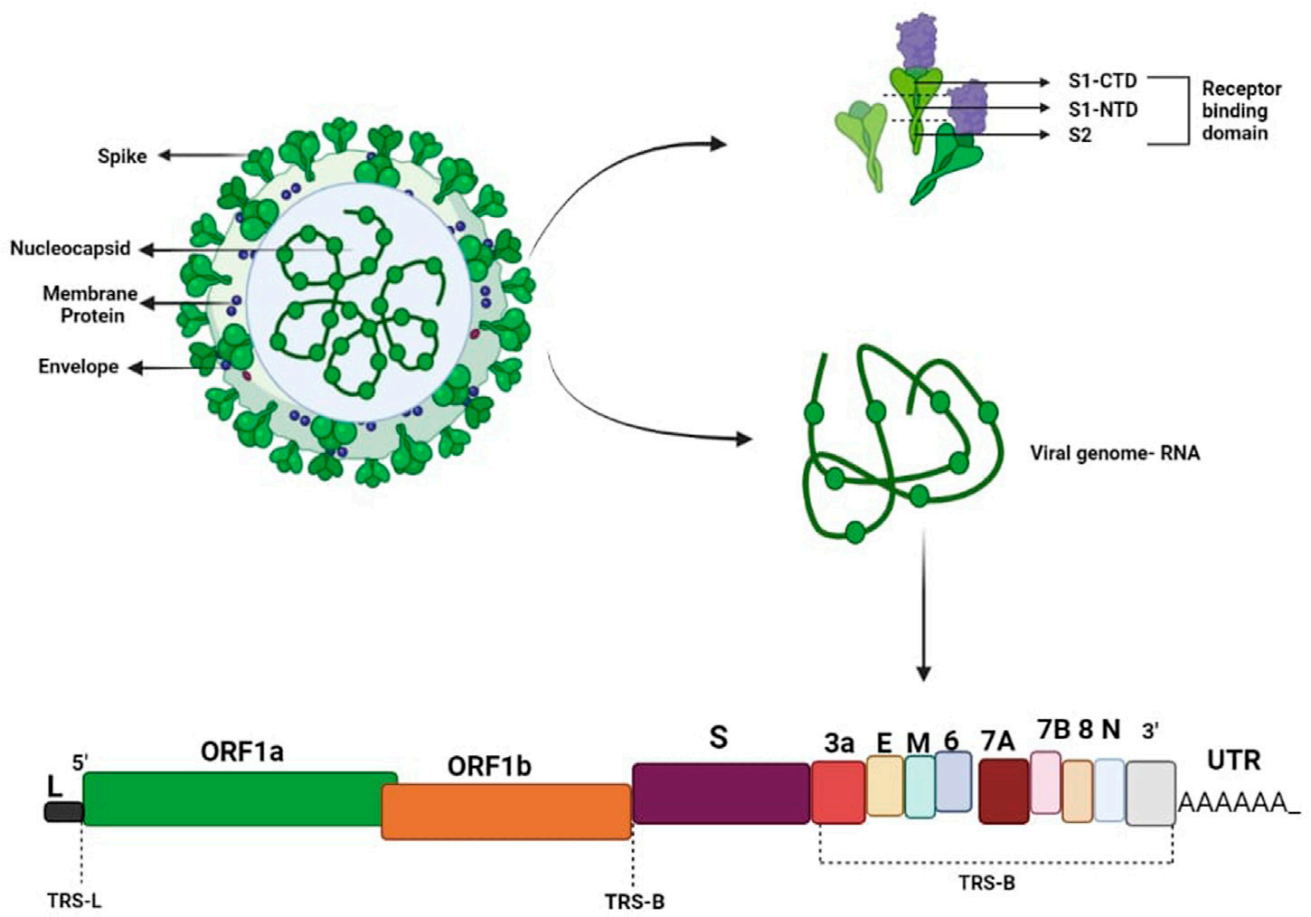
SARS-CoV-2 virus. A functioning polybasic cleavage site at the S1–S2 junction of the spike protein and the receptor-binding domain (RBD) in the S1 subunit are two important genetic traits of SARS-CoV-2.

The SARS-CoV-2 virus, which was first discovered in China, has already spread to every country in the world. The genome of SARS-CoV-2 contains a positive-sensed RNA virus enclosed by an envelope. The genomic sequence of SARS-CoV-2 was first made public in January 2020, and comparisons with other coronaviruses show that it differs from these in terms of its strong affinity for the ACE2 receptor and the presence of a polybasic cleavage site at the S1/S2 spike junction that controls infectivity and host range. After its discovery, various mutations occurred in the virus genome, which resulted in the development of various mutant strains, such as the alpha, beta, and delta variants. The receptor-binding domain (RBD) of the spike (S) protein mediates viral entry by binding with the human cell surface protein angiotensin-converting enzyme 2 (ACE2). Figure 1 shows the structure of SARS-CoV-2. According to sequencing results, the SARS-CoV-2 genome undergoes two single-nucleotide alterations per month. In the alpha variant, 17 mutations can be seen in the genome, which includes mutations of the receptor-binding domain, such as E484K, S494 P, and N501Y, and mutations in the s-glycoprotein comprising 69del, 70del, D614G, 144del, and A570D. These mutations in the receptor-binding domain and s-glycoprotein region make the virus 70% more transmissible. Deletions in the spike protein correlate with the immune response of the infected person. In addition, increased virulence and infectivity have been linked to the N501Y mutation in mouse models (Brief, 2020; Hill et al., 2022). RBD mutations in the beta [K417N/E484K/N501Y] (Planas et al., 2021; Zhou et al., 2021), gamma [K417T/E484K/N501Y] (Burki, 2021; Lancet, 2021; Xie et al., 2021), delta [T478K, and L452R] (Goher et al., 2022), and omicron variants [L452R/F486V/F486V/L452R/L452R, F486V/R493Q] (Mohapatra et al., 2022) have also been explored by scientists. Mutations in the beta variant have been shown to have a stronger affinity (4.62 times higher) for binding hACE2 (Ramanathan et al., 2021). RBD mutations in the epsilon, beta, and theta variants resulted in an increase in infectivity in vitro and also made the virus 20% more transmissible (Ali et al., 2021a; Deng et al., 2021; McCallum et al., 2021; Zhao et al., 2021). In January 2022, a virologist discovered a new variant of SARS-CoV-2 in Cyprus and named it “deltacron.” Deltacron is a super-variant with the combined genome of the delta and omicron variants (Kreier, 2022). Genome analysis of deltacron showed that the RBD is derived from the omicron variant, and this variant may lead to enhanced disease transmission and immune evasion (Colson et al., 2022; Hosch et al., 2022).

### Vaccines for COVID-19

**FIGURE 2:**
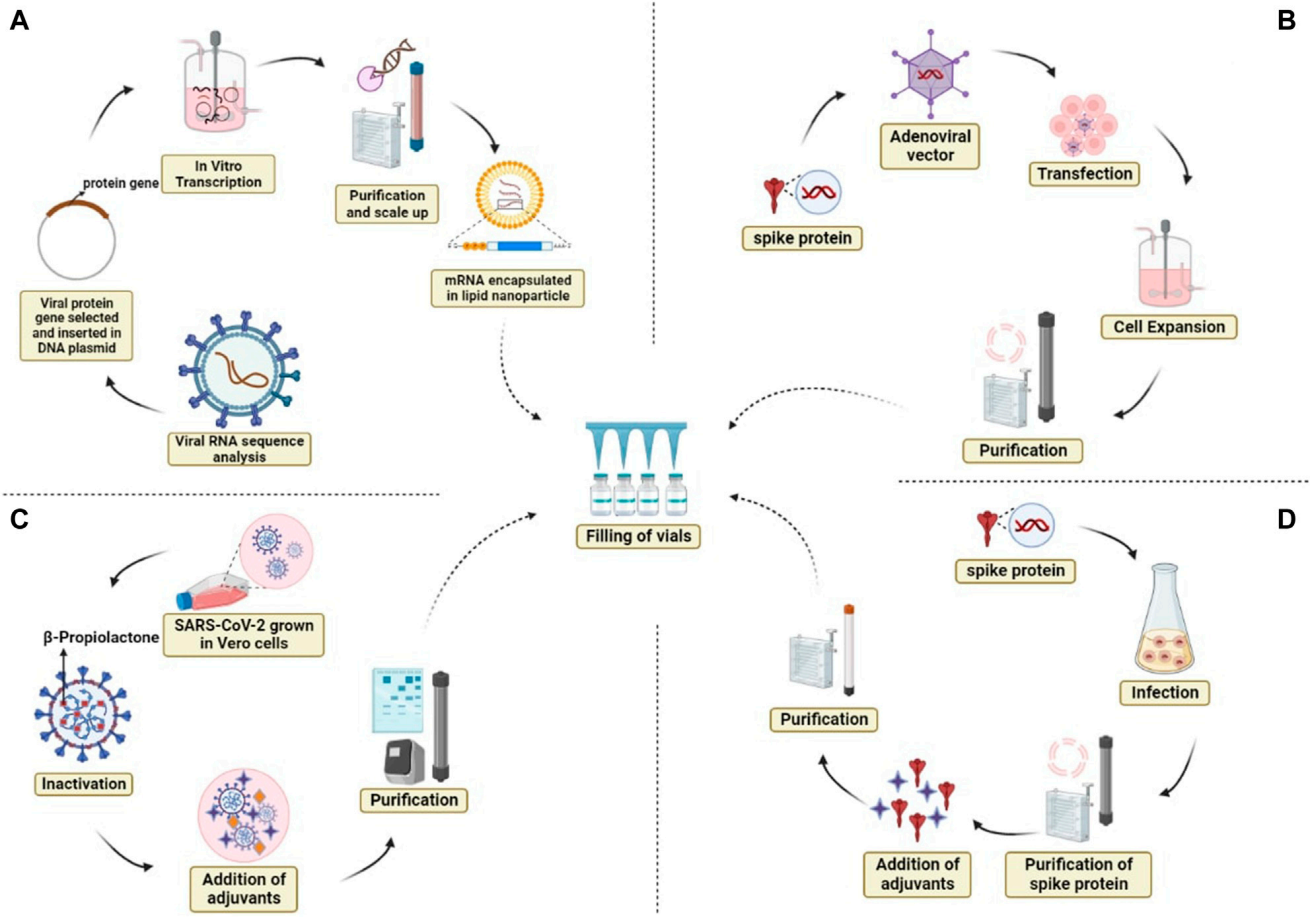
Different platforms for vaccine production and their modes of action. **(A)** mRNA vaccine, **(B)** adenoviral vector vaccine, **(C)** whole inactivated virus, and **(D)** protein subunit vaccine.

The severe effects of COVID-19 on humanity and on every country’s economy raise the need for next-generation vaccine development to eradicate this virus. Through valiant efforts by the scientific community and despite various obstacles, the first COVID-19 vaccine entered human clinical trials in 2020 (Aljabali et al., 2020; Amawi et al., 2020; Mukherjee, 2020; Shereen et al., 2020; Thanh Le et al., 2020; Velavan & Meyer, 2020). After its development, the optimal dosage and schedule was another important aspect that needed to be determined to enhance vaccine efficacy against the infection. Under normal conditions, the vaccine development process requires significant research and testing before the vaccine can be introduced into later-phase clinical trials, but due to the unprecedented circumstances, permission was granted for the emergency use of coronavirus vaccines on the basis of data on their efficacy and safety from early clinical trials (BIO, 2021). However, it is still questionable whether the existing SARS CoV2 vaccinations are safe and effective (Kuppili et al., 2021; Uddin et al., 2021). Because these vaccines were approved on the basis of an emergency situation, proper monitoring of the efficacy, safety, and side effects (if any) of these vaccines is required. Additionally, vaccinations should be evaluated for their efficacy against the several SARS-CoV-2 mutations that have recently emerged. Certain vaccines produce an appropriate immune response after a single dose, whereas others require a booster shot a month or more later. Therefore, a suitable schedule with respect to the gap between the two doses and a booster dose of each vaccine should also be developed to improve their efficacy and results (Torales et al., 2020; Rana et al., 2022). To date, a number of COVID-19 vaccines have been developed and are being globally administered in vaccination programs. Scientists have used different platforms to develop coronavirus vaccines, such as mRNA and proteins*.* The different platforms for vaccine production and their modes of action are shown in Figure 2. An issue that should also be considered during vaccination programs is that vaccine development requires high levels of expertise, extensive infrastructure, and a great deal of money; therefore, low-income countries are not able to produce vaccines. However, to eradicate this virus from Earth, vaccination of the majority of the population is necessary to avoid any future variants, so developed countries must support vaccination programs in these low-income countries (Saied et al., 2022). Based on the platforms used for the production of the coronavirus vaccine, we categorized the vaccines developed into four categories.

A) Nucleic acid-based vaccines (**BNT162b2 and Spikevax**)
B) COVID-19 viral vector/adenovirus vaccines (**JCOVDEN, Vaxzevria, and Sputnik V**)
C) Protein-based vaccines **(Nuvaxovid)**
D) Whole inactivated virus (**BBIBP-CorV, CoronaVac, and Covaxin**)
A) Nucleic acid-based vaccines

Nucleic acid vaccines are genetic vaccines consisting solely of DNA or RNA, which are taken up and translated into proteins by host cells and elicit immune responses. Since naked nucleic acids do not have a viral coat, they are not typically affected by pre-existing immunity, which can reduce the clinical effectiveness of recombinant virus vaccines. Nucleic acid vaccines provide several significant advantages over other forms of vaccination in terms of increased safety and lower production costs (Liu, 2011; Sardesai and Weiner, 2011). Despite the safety concerns regarding DNA/mRNA vaccinations, very little incorporation of viral genes into host genes occurs with the use of plasmid vectors (Sheets et al., 2006). In this article, we compare two major nucleid acid vaccines (BNT162b2 and Spikevax) that have been approved for emergency use.

a) BNT162b2/Comirnaty

The biotechnology companies Pfizer (American) and BioNTech (German) developed an mRNA-based vaccine and named it the Comirnaty/BNT162 vaccine. The SARS-CoV-2 full-length spike protein is encoded by the nucleoside-modified RNA vaccine BNT162b2, which is packaged as a lipid nanoparticle. Preclinical data provided by Pfizer indicated that immunization of a non-primate model (*Rhesus macaques*) with BNT162b2 administered intramuscularly induced the production of neutralizing antibodies and also of T_H_ and T_C_ cells, which protect *R. macaques* from SARS-CoV-2 infection (Khehra et al., 2021). After initial approval by the UK, the vaccine was subsequently also approved by the FDA. Three doses of the BNT162b2 vaccine in children (6 months to 4 years) have been found to produce similar immunity to that produced by two doses in adults (Mayo Clinic, 2021). In children, the vaccine shows different efficacy rates in different age groups (Pfizer, 2020; Pfizer, 2021). The vaccine efficacy for different SARS-CoV-2 variants is as follows: 94% for the alpha variant (Lopez Bernal et al., 2021), 75% for the beta variant (Abu-Raddad et al., 2021), 88% for the delta variant (Lopez Bernal et al., 2021), and 60% for the omicron variant (Tartof et al., 2022). To minimize cases of hospitalization and enhance the efficacy rate, many countries have also immunized their population with a booster dose of BNT162b2 (Edouard et al., 2020).

b) Spikevax/mRNA-1273

The World Health Organization approved Spikevax vaccine, which is manufactured by Moderna, for emergency use against SARS-CoV-2 on 30 April 2021. The spike protein of Coronavirus is encoded by mRNA found in the lipid nanoparticles that make up the Spikevax vaccine. The non-replicating, transiently expressed, transported mRNA is mostly found in dendritic cells and subcapsular sinus macrophages. After recognition by immune cells, this mRNA activates an immune response, which results in the production of B and T cells and thus the protection of an immunized individual from SARS-CoV-2 [Spikevax, 2021; Baden et al., 2021). The efficacy of the Spikevax vaccine has been found to be 51% in children aged 6–23 months, 37% for children aged 2–5 years (Mayoclinic, 2021), and 93.3% for those aged 12–17 years (Verbeke et al., 2021). Immunization with a booster dose has been shown to enhance levels of neutralizing antibodies against the delta variant by 17% (Spikevax, 2021). The efficacy of the vaccine in terms of symptomatic cases and asymptomatic cases has been found to be 94.1% and 63%, respectively (El Sahly et al., 2021; Verbeke et al., 2021). A published study found that the efficacy of two dosages of Spikevax was reduced by 10 times for the delta variant and by more than 100 times in the omicron variant (Macdonald et al., 2022).

B) COVID-19 viral vector/adenovirus vaccines

Viral vector vaccines or Adenovirus vaccines use harmless adenovirus as a vector; this is modified to deliver SARS-CoV-2 genetic material. Immune cells produce antibodies against the protein encoded by this genetic material. In this article, we compare three major viral vector or adenovirus vaccines (JCOVDEN, Vaxzevria, and Sputnik V) that have been approved for emergency use.

a) JCOVDEN/Ad26. COV2-S

JCOVDEN is produced by Janssen Inc., and was approved for emergency use by the World Health Organization on 5 March 2021. A recombinant, non-replicating, human adenovirus type 26 vector that codes for the SARS-CoV-2 spike protein is present in the monovalent vaccine JCOVDEN. JCOVDEN is able to stimulate both neutralizing and S-specific antibodies (Jcovden, 2021). It has not yet been determined whether JCOVDEN is safe and effective for use in children and adolescents (under the age of 18). JCOVDEN is preferably not used over other available vaccines, because this vaccine has been found to cause various side effects, which include hypersensitivity, anaphylaxis, anxiety-related reactions, concurrent illness, and coagulation disorders such as thrombosis with thrombocytopenia syndrome (Jcovden, 2021). The efficacy of this vaccine against symptomatic COVID-19 14 days after vaccination has been found to be 70.1% for the alpha variant and 38% for the beta variant, but its efficiency has been found to be lower for the delta variant. However, the efficacy of the vaccine against severe COVID-19 14 days after vaccination has been found to be 51.1% for the alpha variant and 70.2% for the beta variant. A booster dose of JCOVDEN should be given after 2 months only in people above 18 years of age. The efficacy of a single dose of JCOVDEN has been found to be reduced by 10 times for the delta variant and by more than 100 times for the omicron variant (Macdonald et al., 2022).

b) Vaxzevria/ChAdOx1-S/Covishield

The British–Swedish multinational pharmaceutical and biotechnology business AstraZeneca produces Vaxzevria, which is commonly known as ChAdOx1-S. In addition, the Serum Institute of India produces Covishield. Vaxzevria contains a replication-deficient chimpanzee adenovirus that encodes for the coronavirus spike glycoprotein. One dose of the vaccine contains 2.5 × 10^8^ infectious units (If. U) created by recombinant DNA technology and genetically altered HEK 293 cells (Vaxzevria, 2021). The dosage gap between the primary and secondary doses should be 4–12 weeks, as recommended by officials (Vaxzevria, 2021). To date, this vaccine has not been approved for the pediatric population, so its efficacy rate in this population is not known. The side effects of Vaxzevria reported so far include hypersensitivity, anaphylaxis, anxiety-related reactions, concurrent illness, and coagulation disorders, such as thrombosis with thrombocytopenia syndrome (Vaxzevria, 2021). The efficacy of Vaxzevria vaccine has been found to be 74.0% for symptomatic cases (Vaxzevria, 2021) and 54% for asymptomatic cases (Pramod et al., 2022). A booster dose should be administered at least 3 months after the secondary dose. The efficacy of the vaccine after the primary dose is slightly lower for the delta variant of the virus (71%) compared to the alpha strain (76%) (Lopez Bernal et al., 2021; Stowe et al., 2021).

c) Sputnik V

The Russian institute Gamaleya National Research Institute of Epidemiology and Microbiology developed Sputnik V based on two different human adenovirus vectors, for adenovirus 26 and adenovirus 5 (Jones and Roy, 2021). Both components of this vaccine (Adenovirus 26 and Adenovirus 5) contain the spike protein gene of SARS-CoV-2. Both components of the vaccine are administered in the form of two doses separated by 3 weeks (Cdsco, 2021). The vaccine induces humoral and cellular immunity against infection caused by SARS-CoV-2. The mechanism of the drug’s action is based on the ability of Ad26- and Ad5-based recombinant viral particles carrying the SARS-CoV-2 S protein gene to efficiently transduce the cells of the vaccinated body; in this case, genetic sequences that code the antigen are delivered to the cells so that the transduced cells start to produce the antigen. After the first dose, the rAd26-based vector enters the body cells, which leads to the expression of SARS-CoV-2 S protein and triggers the development of SARS-CoV-2 immunity. The rAd5-based vector targets body cells following the second dose of the vaccine and strengthens protective immunity toward SARS-CoV-2. Data on the efficacy of Sputnik V are not available for the pediatric population because this vaccine is not approved for the vaccination of children. Chills, fever, headaches, soreness at the injection site, and other adverse reactions are possible with this vaccine. The efficacy of the vaccine has been found to be 73.1% after the primary dose and 91.6% after the secondary dose (Logunov et al., 2021). The efficacy of the vaccine against the alpha and delta variants after the primary dose has been reported as 85.7% (Vokó et al., 2022) and 78.6% (González et al., 2021), respectively. There is an 8.1-fold decrease in neutralizing antibody titers for the omicron version, according to a study published in *Vaccines* (Lapa et al., 2022). According to statistical data made public by the Ministry of Health of the UAE, the vaccine was found to have 97.8% efficacy in averting symptoms of COVID-19 and 100% efficacy in preventing severe illness in 81,000 people who had received two doses (Precision Vaccinations, 2022). In a study involving 40,387 adults aged 60 to 79 who were vaccinated and 146,194 individuals who were not, the Buenos Aires Health Ministry in Argentina found that a single dose of Sputnik Light reduced symptomatic infections by 78.6%, hospitalizations by 87.6%, and deaths by 84.7% (Bianca Nogrady, 2021).

C) Protein-based vaccines

SARS-CoV-2 protein is used in protein-based vaccines; whenever this protein is detected by a person’s immune system, an immune response is produced. A whole protein, protein fragment, or peptide can be used to make protein-based vaccines. The only significant protein-based vaccine that has been authorized for emergency use is Nuvaxovid.

a) Nuvaxovid

Nuvaxovid is a protein-based vaccine for coronavirus manufactured by Novavax, Inc. Nuvaxovid is composed of the full-length recombinant spike protein of SARS-CoV-2, which is adjuvanted with Matrix-M (Fractions A C of *Quillaja saponaria Molina* extract) (Nuvaxovid, 2021). Matrix-M adjuvantation helps in the enhancement of the innate immune response and activation of B and T cells in response to the s-protein. In India, Novavax Inc. has collaborated with the Serum Institute of India to market the vaccine as Covovax. The European Medicines Agency has granted permission for the emergency use of Nuvaxovid in Europe. The efficacy of Nuvaxovid has been found to be higher in children (12–17 years old) compared to adults. Overall, the efficacy of the vaccine has been found to be 89.7% (Heath et al., 2021).

D) Whole inactivated virus-based vaccines

Whole inactivated virus-based vaccines use a killed or inactivated COVID-19 virus strain; when this killed virus is recognized by immune cells, an immune response is produced. In this article, we compare three major whole inactivated virus-based vaccines (BBIBP-CorV, CoronaVac, and Covaxin) that have been approved for emergency use.

a) BBIBP-CorV

The BBIBP-CorV vaccine is manufactured by Sinopharm, a company located in Beijing, China. The BBIBP-CorV vaccine of the whole inactivated virus type. This type of vaccine contains a virus whose genetic material has been damaged by radiation, heat, or chemicals, but that still possesses the ability to induce an immunological reaction. BBIBP-CorV is produced using an aggressive WIV04 strain of SARS-CoV-2 inside Vero cells. The virus is inactivated by beta-propiolactone while the integrity of other viral particles is maintained. Aluminum hydroxide is used as an adjuvant and combined with the resultant inactivated virus to increase the immune response against viral particles. After initial approval by China, the World Health Organization approved this vaccine for emergency use throughout the world (Xia et al., 2020). A 21-day gap between the two doses has been demonstrated to induce the production of a high level of neutralizing and SARS-CoV-2-specific IgG antibodies. The efficacy of this vaccine against symptomatic COVID-19 infection was shown to be 79% in an international phase III trial (Xia et al., 2020). According to reports, a booster dose of the BBIBP-CorV vaccine causes a 1.5–5-fold increase in neutralizing antibodies against omicron compared to a two-dose regime (Ewunkem et al., 2022; Wang et al., 2022).)

b) CoronaVac

Sinovac, which is a China-based company, developed CoronaVac using a whole inactivated virus method. The active ingredient in CoronaVac is an inactivated CZ02 strain (SARS-CoV-2 Virus strain), and aluminum hydroxide is used as an adjuvant to enhance the immune response (WHO, 2021a). A phase III trial of the vaccine conducted in Brazil showed 50.7% efficacy against symptomatic infection, while efficacy of 100% was observed in the prevention of severe cases and hospitalization (Leung et al., 2022). In a population of 3- to 5-year-old children, efficacy against symptomatic cases, hospitalization, and severe illness was found to be 38.2%, 64.6%, and 69.0%, respectively (Florentino et al., 2022). The efficacy of the vaccine in 6- to 11-year-olds is 41.5% and 63.5% against symptomatic and severe cases, respectively (Cheng et al., 2022). In China and Hong-Kong, the vaccine has been approved for use in the pediatric population despite insufficient data. In adults, two doses should be administered at an interval of 28 days for improved efficacy.

c) Covaxin

**FIGURE 3:**
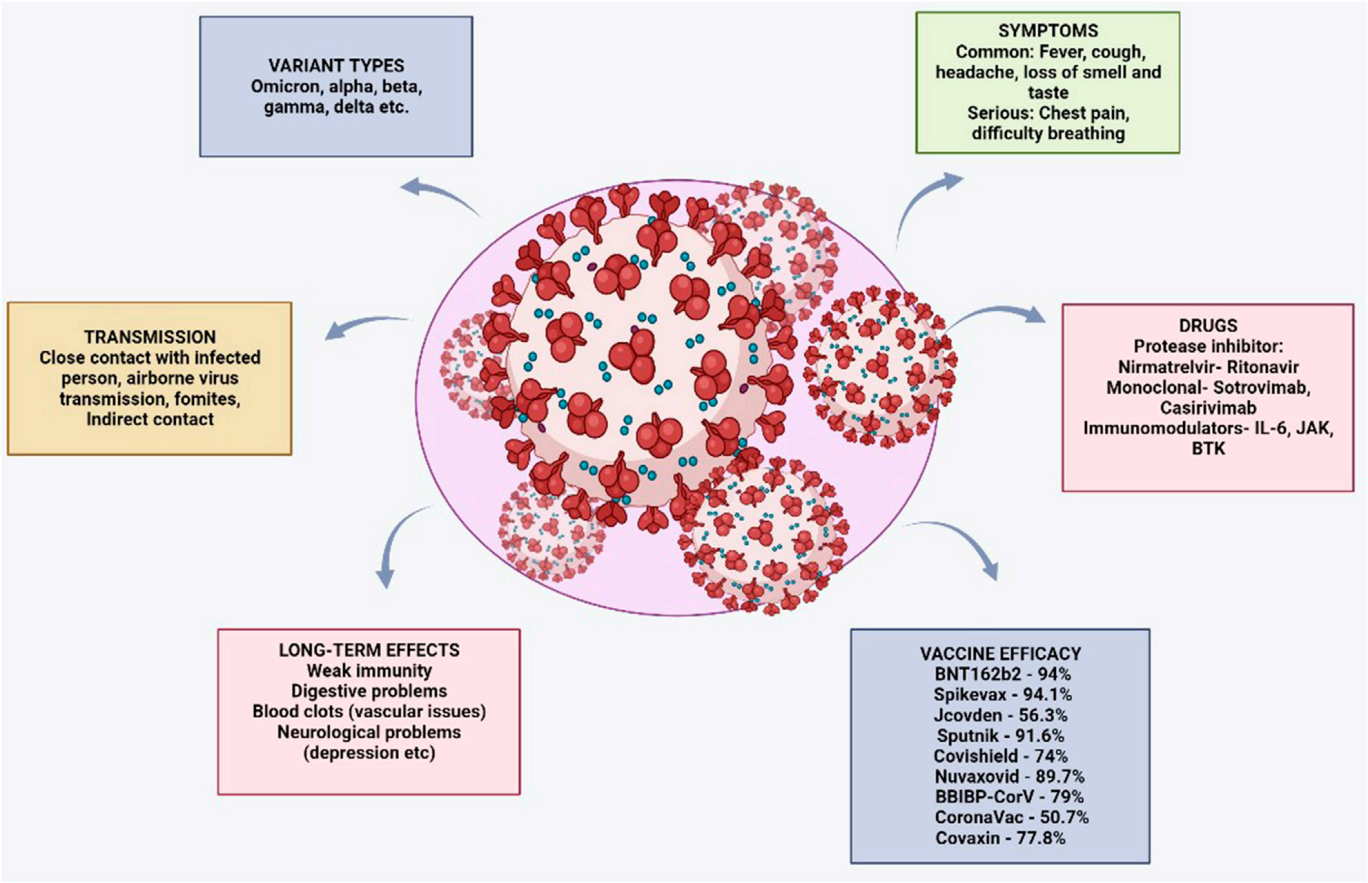
Comprehensive overview of the coronavirus, including relevant risk factors and drugs used for its treatment.

Bharat Biotech produces Covaxin in partnership with the National Institute of Virology (ICMR). The vaccine is manufactured using the beta-propiolactone inactivated strain (Asp614Gly) of SARS-CoV-2. To enhance the immune response, Alhydroxiquim-II (financed by the National Institute of Health) is used as an adjuvant. In India, the vaccine was granted approval for emergency use in November 2021 and for full use in January 2022 by the Drugs Controller General of India (DGCI). In a phase III trial of Covaxin, efficacy was reported to be 77.8% against symptomatic cases and 63.6% against asymptomatic cases (Bharat Biotech, 2021; Yadav et al., 2022). The levels of neutralizing antibodies against omicron produced by two doses of the vaccine are frequently insufficient, demonstrating its weak ability to trigger immune responses against omicron. In a phase II/III trial of Covaxin in children (2–18 years old), the vaccine was found to be safe and to induce a sufficient immune response with no extreme side effects (Sapkal et al., 2022). Figure 3 shows a comprehensive overview of the coronavirus, including its risk factors and the drugs used for treatment.

As discussed above, various types of vaccines provide different efficacy rates, and their efficacy also varies for different variants of SARS-CoV-2. Therefore, a comparison is needed to determine the best available vaccine for each variant. Table 1 shows a comparison of the different types of widely used vaccines worldwide.

**TABLE 1:** Comparison of the different types of widely used vaccines worldwide.

S. No	Vaccine name and type	Company and manufacturing date	Efficacy	Cost	Recommended dose	Countries approved	References
1	BNT162b2/Comirnaty (mRNA vaccine)	Pfizer and BioNTech (31 December 2020)	Alpha, 94%; beta, 75%; delta, 88%; omicron, 60%	EU and United States of America: $19.50	Two doses (21-day gap)	The United States of America, Austria, Brazil, *etc.*	Kuppili et al. (2021); Lancet (2021); Lapa et al. (2022)
2	Spikevax/mRNA-1273 (mRNA vaccine)	Moderna (30 April 2021)	94.1% (symptomatic cases); 63% (asymptomatic cases)	EU: $25.5 United States of America: $15 Argentina: $21.5 Botswana: $28.8	Two doses (28-day gap)	European Union, Japan, Austria, the US, the United Kingdom, *etc.*	Leung et al. (2022); Libster et al. (2021)
3	JCOVDEN/Ad26.COV2-S (adenovirus vaccine)	J&J/Janssen (5 March 2021)	56.3% (symptomatic cases); 34.2% (asymptomatic cases)	EU: $8.5 United States of America: $10 African Union: $10	One dose	India, European Union, Colombia, the United States of America, Brazil, *etc.*	Liu (2011)
4	Vaxzevria/ChAdOx1-S/Covishield (adenovirus vaccine)	AstraZeneca and the Serum Institute of India (16 February 2021)	74% (symptomatic cases); 54% (asymptomatic cases)	EU: $2.15	Two doses (4–12-week gap)	Europe, Africa, America, India, Australia, the United Kingdom, and the United States of America	Lopez Bernal et al. (2021); Luke et al. (2010)
5	Sputnik V (adenovirus vaccine)	Gamaleya National Research Institute of Epidemiology and Microbiology (12 April 2021)	91.6% (symptomatic cases)	<$10	Two doses (21-day gap)	Argentina, Serbia, the United States of America, India, UAE, *etc.*	Maulud et al. (2022)
6	Nuvaxovid (protein vaccine)	Novavax (20 December 2021)	89.7% (symptomatic cases)	Denmark: $20.9	Two doses (21-day gap)	India, Turkey, Malaysia, Denmark, *etc.*	Nguyen et al. (2022)
7	BBIBP-CorV (whole inactivated virus)	Sinopharm/Beijing Institute of Biological Products (April 2021)	79% (symptomatic cases)	Argentina, Mongolia: $15 Senegal: $18.6 China: $30 Hungary: $36	Two doses (28-day gap)	Asia, Africa, South America, Argentina, China, *etc.*	Nordström et al. (2022)
8	CoronaVac (whole inactivated virus)	Sinovac Biotech (1 June 2021)	50.7% (symptomatic cases); 100% (hospitalization)	China: $29.75 Ukraine: $18 Philippines: $14.5 Brazil: $10.3 Cambodia: $10	Two doses (28-day gap)	China, Indonesia, Singapore, *etc.*	Perotti et al. (2020)
9	Covaxin (whole inactivated virus)	Bharat Biotech in collaboration with the National Institute of Virology (ICMR) (3 November 2021)	77.8% (symptomatic cases); 63.6% (asymptomatic cases)	$3–$4	Two doses (28-day gap)	India, Brazil, Iran, Mexico, *etc.*	Planas et al. (2021); Polack et al. (2020)

### Convalescent plasma therapy

Convalescent plasma therapy (CPT) was discovered in the past as a therapy for other respiratory viral diseases, such as MERS-CoV-2 (Luke et al., 2006; Mair-Jenkins et al., 2015); it was found that this therapy results in the generation of a temporary immune response and reduction in viral particles in the infected individual, which further helps in the avoidance of cytokine storm (Luke et al., 2010; Arciuolo et al., 2017). The major advantage of CPT is that it is readily available as soon as an individual who has recovered from the infection becomes available, but it does require further development. Despite certain drawbacks, such as the need for dedicated collection, testing, dose standardization, and blood group testing, this therapy serves as a first line of defense (Luke et al., 2010). Despite being poorly defined and associated with some controversy, CPT is among the therapeutic strategies that are under investigation for efficacy against SARS-CoV-2 (Duan et al., 2020; Focosi et al., 2020; Perotti et al., 2020; Focosi and Farrugia, 2021). The FDA granted permission for the emergency use of COVID-19 convalescent plasma on 23 August 2020 for the treatment of COVID-19 patients who were taking immunosuppressive medications. Various studies have attempted to explore the role of CPT in individuals infected with SARS-CoV-2. Some of these have found that CPT plays an important role in enhancing the survival rate of patients (Agarwal et al., 2020; Klassen et al., 2021), whereas others have found that CPT does not play any significant role in survival rate, especially in severe cases (Janiaud et al., 2021). These findings created confusion for the public, scientists, and governments regarding the efficacy and safety of CPT, prompting Daniele Focosi et al. to conduct a review of several randomized clinical trials (RCTs); they found that these RCTs produced different results for several reasons, such as CPT dose and timing (Focosi et al., 2022). Reports suggest that the benefits of CPT are increased when it is administered with high neutralizing antibody titers within 3 days of the onset of symptoms (Libster et al., 2021; González et al., 2022; Paneth et al., 2022). The main issue in CPT is the administration of high-quality CPT by transfusion. Although CPT titer ≥1:320 is recommended for use in immunocompromised people, its effective/optimum dose and the timing of administration should be assessed in further clinical trials (Focosi and Franchini, 2021; Franchini et al., 2021).

### Chance of reinfection

Beyond the efficacy of vaccines, the most important question is to determine the chances of reinfection with the same or a different SARS-CoV-2 variant after a patient has recovered. Patients who have recovered from COVID-19 appear to have memory B and T cells, in accordance with findings indicating that infection with SARS-CoV-2 generates both a neutralizing antibody response and a cellular response with virus-specific T cells (Wajnberg et al., 2020a; Juno et al., 2020; Le Bert et al., 2020). Approximately a week after developing symptoms, more than 90% of those with SARS-Cov2 develop antibodies that persist for at least 3 months (Wajnberg et al., 2020b; Gudbjartsson et al., 2020). However, antibody titers may eventually decrease in cases of mild illness (Ibarrondo et al., 2020). It is critical to gain a better understanding of whether COVID-19 survivors are immune to reinfection or not. Reinfection occurs when a person contracts an infection once, recovers, and then contracts it again—either from the same infectious agent or a different variation (Yahav et al., 2021). According to the CDC, recovered patients should have at least one negative PCR test result for SARS-Cov-2. In 2021, the World Health Organization stated that the presence of antibodies in patients after recovery from COVID-19 does not guarantee protection from reinfection (WHO, 2021b). The reinfection rate in different countries has been reported to range from less than 0.5% to more than 5% [117,118]. Different studies have confirmed cases of reinfection with mild-to-moderate symptoms in the second infection, depending on the time interval between the primary and secondary infections and the level of detectable IgG against SARS-CoV-2 (Ali et al., 2021b; Breathnach et al., 2021; Caralis, 2021; Hall et al., 2021; To et al., 2021; Nguyen et al., 2022; Sotoodeh Ghorbani et al., 2022). A study conducted in Sweden found that the risk of SARS-CoV-2 reinfection and COVID-19 hospitalization in individuals who have survived and recovered from a previous infection remained low for up to 20 months (Nordström et al., 2022).

### Herd immunity

The major drawback of the possibility of reinfection is the reduced chance of herd immunity of the population. Herd immunity means that a sufficient percentage of immune people are present in a population to achieve the indirect protection of vulnerable people from infection. Herd immunity is important for the protection of individuals who cannot be vaccinated, including the very young and the immunocompromised. Reinfection can increase the chances of contact between infected individuals and susceptible hosts. According to various studies, the reproductive number of SARS-CoV-2 is estimated to fall within the range of 2.2 to 5.7 (Li et al., 2020; Sanche et al., 2020), whereas herd immunity is achieved when the reproductive number is less than 1. Evidence suggests that the spread of SARS-CoV-2 will not cease until at least 50% of the population has developed immunity. Given that SARS-CoV-2 has a very high case fatality rate, infection of 50% of the population would lead to a significant number of deaths (Flaxman et al., 2020; Salje et al., 2020). Therefore, vaccines may be a promising way of reaching herd immunity. However, vaccine hesitancy due to fear of side effects, religious beliefs, and misinformation about vaccines is a major hurdle that inhibits the attainment of herd immunity in the population (Wong, 2021).

### Future directions

The SARS-CoV-2 pandemic has brought into focus unexpected and significant issues for humanity. Numerous containment techniques, including the utilization of genetic and community monitoring, and an increase in immunization and the provision of booster doses to the susceptible population, have been developed to reduce the harmful effects associated with the many forms of SARS-CoV-2 (Dhawan et al., 2022). Although a number of proteomic techniques are available for detection of the virus, more techniques with higher specificity should be sought (Rana et al., 2020). The pandemic has forced us to explore different existing viruses by integrating artificial intelligence and machine learning, as these viruses could potentially pose a threat to humans in the future. We cannot ignore the possibility that a completely new virus could emerge and induce another global pandemic in the future. According to studies, those who are not immunized are more prone to experience serious illness, leading to hospitalization. Based on prior experiences with other viruses, we understand that further information regarding the transmissibility of the virus, the effectiveness of vaccination, and the severity of illness caused by coronavirus will only be available with time and careful monitoring. Meanwhile, studies should focus on the development of a vaccine that is equally effective for all variants of the virus.

### For the research community

The research community should focus on developing a vaccine that is equally effective against all variants. Furthermore, effective methods of detecting virus variants as soon as possible should be developed so that proper measures can be taken to prevent the spread of the disease (Maulud et al., 2022; Rana et al., 2022). Additionally, previous studies have shown that antibody levels are reduced 3–4 months after vaccination (Polack et al., 2020; Khoury et al., 2021; Naaber et al., 2021). Along with vaccines, other compounds should be tested for their role in the treatment of COVID-19. For example, studies have shown that use of 2% hydrogen as a line of treatment can enhance patient immunity and may result in an exceptional decrease in toxicity and oxidation processes, as hydrogen displays various antioxidant, anti-inflammatory, and anti-apoptosis properties (Yang et al., 2020). In support of this, various experiments have been performed to verify the effects of hydrogen in an animal model; these have shown that after inhalation of 2% hydrogen by cerebral ischemia-reperfused rats, their condition is improved, with a high positive recovery rate (Channappanavar and Perlman, 2017). This mechanism works by selective elimination of hydroxyl radicals and peroxynitrite anions by H_2_. This technique also helps to lower cytokine levels (TNF-α, IL-2, IL-7, and IL-10) in COVID-19 patients. Through the use of hydrogen inhalation as a treatment, lung injury can also be prevented (Waqas et al., 2020). Additionally, COVID-19 patients often experience organ failure, which can lead to death. Organ failure occurs due to an unstable internal environment in the body and increased levels of malondialdehyde in the lungs, along with other toxic substances. Clinical therapy with hydrogen can help with the activation of the antioxidant enzyme superoxide dismutase, which helps in eliminating toxic substances and damaged DNA from the body; it also stabilizes the internal environment, which enhances defense mechanisms in patients (Rana et al., 2020). Crystallographic analysis of class I MHC/peptide complexes has shown that the majority of charged peptide core residues are exposed in MHC complexes and are recognized by T-cell receptors (Rouzbahani et al., 2022). By boosting host immunogenicity, an appropriate multi-epitope vaccine can support the immune response and thus reduce the chance of reinfection (Rouzbahani et al., 2022; Sajid et al., 2022). It is crucial to identify T-cell epitopes quickly and accurately, but doing so can considerably reduce the amount of experimental labor required in carrying out culturing to determine the *in vitro* expression required to develop vaccines based on these epitopes. Additionally, repurposing of available drugs could also be an effective method for elimination of this infection.

### For governments, NGOs, and the public

Governments and non-governmental organizations can also play an important role in generating awareness and in the elimination of ethical and religious myths regarding the vaccine. They can also provide funding to the research community so that money is not an limiting factor in the development of vaccines. Additionally, governments of all countries should focus on the vaccination of people all over the world, as vaccination of the maximum possible proportion of the global population is necessary to avoid the next variants of SARS-Cov-2 (Islam et al., 2022; Mattiuzzi and Lippi, 2022) and to achieve herd immunity. Governments should heed the WHO slogan that ‘none of us is safe until all of us are safe.’ Furthermore, a proper channel should be established for faster approval of effective vaccines. The public can also play a major role in the prevention of other variants of this virus. Everyone should get vaccinated, and anyone who has any doubts regarding the production and efficacy of the vaccine should resolve these doubts by referring to the relevant sources. The public should also follow the guidelines provided by the government at times, as well as complying with social distancing, wearing a mask, following good hygienic practices, and avoiding social gatherings during pandemics.

## Conclusion

Since its discovery, coronavirus has spread across the globe and emerged as a significant public health threat. At this point in the pandemic, with debate underway regarding vaccine efficacy and drug repurposing, a comparative study of vaccine efficacy is much needed to combat this disease. Screening, infection prevention, quarantining of ill people, and preventive self-isolation of contacts are crucial steps to reduce the number of new cases. The discovery of new variants each day serves as a sobering reminder that the world is still dealing with a pandemic and that another SARS-CoV-2 outbreak could happen at any time. The available vaccines should be compared on the basis of their efficacy against the different variants of the virus, and authorities should develop appropriate and strict guidelines to ensure the survival of humankind. Beyond vaccines, the scientific community should also focus on the use of convalescent plasma therapy for therapeutic purposes, as this could be very promising for low-income countries where vaccine production for a large population is not possible. The achievement of herd immunity requires vaccination of the wider population for the protection of immunocompromised and susceptible individuals. Therefore, the vaccination process should be accelerated and disruption should be avoided. Scientists should also examine the production of antibodies with the use of a combination of vaccines; their efficacy should be tested; and the chances of reinfection should also be studied. The public must maintain a high level of caution when hosting social events and make sure that everyone who qualifies has received all necessary vaccinations, including the third and/or booster dose. Finally, it is the duty of all of us, and not only governments or the scientific community, to combat this disease.

## Abbreviations

SARS-CoV-2, severe acute respiratory syndrome coronavirus 2; COVID-19, coronavirus disease 19; ACE 2, angiotensin-converting enzyme 2; WHO, World Health Organization; CDC, Centers for Disease Control and Prevention; PCR, polymerase chain reaction; RBD, receptor-binding domain; If. U, infectious units; CPT, convalescent plasma therapy; MERS-CoV-2, Middle East respiratory syndrome coronavirus; MHC, major histocompatibility complex.
